# Risk factors for postoperative acute kidney injury after cardiac surgery

**DOI:** 10.47487/apcyccv.v6i3.491

**Published:** 2025-09-24

**Authors:** Ignacio M. Piaggio, Juan F. Furmento, Juan P. Costabel, Daniel Navia, Francisco A. Olivares, Alan Sigal, Mariano Vrancic, Fernando Piccinini, Leonardo A. Seoane

**Affiliations:** 1 Instituto Cardiovascular de Buenos Aires (ICBA), Buenos Aires, Argentina. Instituto Tecnológico Buenos Aires Instituto Cardiovascular de Buenos Aires (ICBA) Buenos Aires Argentina; 2 Servicio de Cardiología Crítica, Departamento de Cardiología. Instituto Cardiovascular de Buenos Aires (ICBA), Buenos Aires, Argentina. Servicio de Cardiología Crítica Departamento de Cardiología Instituto Cardiovascular de Buenos Aires (ICBA) Buenos Aires Argentina; 3 Servicio de Cirugía cardiovascular, Instituto Cardiovascular de Buenos Aires (ICBA), Buenos Aires, Argentina. Servicio de Cirugía cardiovascular Instituto Cardiovascular de Buenos Aires (ICBA) Buenos Aires Argentina; 4 Servicio de Cardiología Clínica, ICBA Instituto cardiovascular de Buenos Aires, Buenos Aires, Argentina. Servicio de Cardiología Clínica ICBA Instituto cardiovascular de Buenos Aires Buenos Aires Argentina

**Keywords:** Acute Kidney Injury, Cardiac Surgery, Risk Factors, Extracorporeal Circulation, Mortality, Injuria Renal Aguda, Cirugía Cardíaca, Factores de Riesgo, Circulación Extracorpórea, Mortalidad

## Abstract

**Objectives.:**

To describe the frequency of postoperative acute kidney injury (AKI) following cardiac surgery and to analyse risk factors for its development.

**Materials and Methods.:**

We conducted an observational analytical cohort study using prospective data from adult patients who underwent cardiac surgery between 2003 and 2023 at a high-complexity centre in Argentina. AKI was defined according to KDIGO criteria. Risk factors were assessed using logistic regression.

**Results.:**

A total of 13,215 patients were analysed, with a mean age of 64 years; most were men (75.7%). The frequency of AKI was 7.3%, with an in-hospital mortality of 4.7%. Independent risk factors included advanced age (OR: 1.05, p<0.001), urgent surgery (OR: 2.87, p<0.001), cardiopulmonary bypass (OR: 1.41, p<0.001), and comorbidities such as chronic obstructive pulmonary disease and preoperative anaemia. Asymptomatic status prior to surgery was identified as a protective factor.

**Conclusions.:**

The frequency of postoperative AKI is comparable to international registries. The main predictors of risk were age, surgical urgency, and use of cardiopulmonary bypass. Identifying risk factors can improve perioperative prevention and management in cardiac surgery.

## RESUMEN

**Objetivos.:**

Describir la frecuencia de insuficiencia renal aguda (IRA) posoperatoria en cirugía cardíaca y analizar los factores de riesgo para su desarrollo.

**Materiales y métodos.:**

Se realizó un estudio de cohorte observacional y analítico, utilizando datos prospectivos de pacientes adultos sometidos a cirugía cardíaca entre 2003 y 2023 en un centro argentino de alta complejidad. La IRA se definió según criterios KDIGO. Los factores de riesgo se evaluaron mediante regresión logística.

**Resultados.:**

Se analizaron 13 215 pacientes con una edad promedio de 64 años, predominando varones (75,7%). La frecuencia de IRA fue del 7,3%, con una mortalidad intrahospitalaria del 4,7%. Los factores de riesgo independientes incluyeron edad avanzada (OR: 1,05, p<0,001), cirugía de urgencia (OR: 2,87, p<0,001), circulación extracorpórea (OR: 1,41, p<0,001) y comorbilidades como enfermedad pulmonar obstructiva crónica y anemia preoperatoria. Como factor protector se observó el estado asintomático previo a la cirugía.

**Conclusiones.:**

La frecuencia de IRA posoperatoria es similar a los registros internacionales. Los principales factores predictores de riesgo fueron la edad, la electividad de la cirugía y el uso de bomba de circulación extracorpórea. La identificación de factores de riesgo permite mejorar la prevención y manejo perioperatorio en cirugía cardíaca.

## Introduction

Acute kidney injury (AKI) is a major complication after cardiac surgery, associated with increased morbidity, mortality, prolonged hospital stays, and higher healthcare costs. ^1^^-^^3^^)^ International reports estimate the incidence of postoperative AKI (PO-AKI) at 20-30%, underscoring the importance of early identification of patients at risk. ^4^^,^^5^ Although the need for dialysis is less frequent, it is linked to a sharp rise in mortality, highlighting the relevance of preoperative preventive measures. ^6^

In Argentina, registries such as CONAREC XVI have shown that AKI frequency varies according to institutional factors and access to preventive strategies, reporting an overall incidence of 13.3%. ^7^ More recent data from the ARGEN-CCV registry documented PO-AKI rates of up to 12.1% among patients undergoing coronary artery bypass grafting (CABG). ^8^

The development of AKI after cardiac surgery is multifactorial, involving preoperative, intraoperative, and postoperative determinants. Despite advances in surgical techniques and renal protection strategies, the incidence remains substantial, and no specific preventive therapy has been established. ^4^^,^^5^^,^^9^ Ongoing studies are exploring targeted interventions in the inflammatory cascade, including complement inhibition. ^10^^-^^12^

The aim of this study was to describe the frequency of PO-AKI in cardiac surgery at a high-complexity center in Argentina, to identify risk factors for this complication, and to assess its impact on in-hospital mortality and the need for hemodialysis.

## Materials and methods

### Study design

We conducted an observational analytical cohort study using the prospectively maintained database of the Cardiac Surgery Department, Instituto Cardiovascular de Buenos Aires (ICBA), between November 2003 and March 2023.

### Study population

Consecutive adult patients (≥18 years) undergoing cardiovascular surgery with or without cardiopulmonary bypass (CPB) were included. Patients with chronic kidney disease, defined as an estimated glomerular filtration rate (eGFR) <60 mL/min per 1.73 m² for more than 3 months, were excluded. As all participants were adults, no age-related exclusions were applied.

### Variables and definitions

Sociodemographic variables (age, sex), clinical variables (hypertension, diabetes, dyslipidemia, smoking, previous myocardial infarction, chronic obstructive pulmonary disease [COPD], stroke, preoperative anemia, heart failure as clinical presentation, ventricular dysfunction, asymptomatic status), and surgical variables (urgency, type of surgery, use of CPB, reoperation) were collected. Age was recorded in years as a continuous variable. Sex and clinical variables were analyzed as dichotomous variables. Smoking was defined as current daily smoking or smoking within the previous 12 months.

PO-AKI was defined according to Kidney Disease: Improving Global Outcomes (KDIGO) criteria: stage 2, plasma creatinine more than twice baseline within 7 days plus oliguria (<0.5 mL/kg per h for more than 12 h); and stage 3, plasma creatinine more than three times baseline or dialysis requirement with oligoanuria (<0.3 mL/kg per h for more than 24 h or anuria for 12 h) ^13^. AKI was analyzed as a dichotomous variable. Preoperative anemia was defined as hemoglobin <12 g/dL in women and <13 g/dL in men within 1 month before surgery and analyzed as a dichotomous variable. Heart failure as a clinical presentation was defined as signs and symptoms of congestion (orthopnea, paroxysmal nocturnal dyspnea, dyspnea in New York Heart Association [NYHA] functional class II or higher, hepatomegaly, third heart sound, lower-limb edema, or bibasilar crackles) confirmed by Doppler echocardiography showing increased filling pressures. Asymptomatic patients were those with no dyspnea, angina, palpitations, dizziness, syncope, or hospitalization for heart failure in the 3 months preceding surgery. Ventricular dysfunction was defined as left ventricular ejection fraction ≤40% by the biplane Simpson method on Doppler echocardiography and analyzed as a dichotomous variable

### Statistical analysis

Quantitative variables were expressed as mean ± standard deviation (SD) or as median and interquartile range (IQR), according to distribution, and categorical variables as percentages. Data normality was assessed with the Kolmogorov-Smirnov test. The chi-square test was used for categorical variables, and Student’s t-test or the Mann-Whitney U test was applied for continuous variables, depending on whether the distribution was parametric or non-parametric.

Associations were first examined in univariate analyses and subsequently in multivariate logistic regression models adjusted for clinically relevant variables. Variables selected for bivariate analysis were those considered prognostically important in cardiac surgery, according to the literature and clinical judgment. (5,9) Variables significant in univariate analysis (p<0.05) were entered into the multivariate models. Odds ratios (OR) and 95% confidence intervals (CI) were reported. A p-value <0.05 was considered statistically significant. Analyses were done with SPSS version 29.0.

### Ethical aspects

The study was conducted in accordance with the Declaration of Helsinki and was approved by the Clinical Research Committee of the Instituto Cardiovascular de Buenos Aires (ICBA) and its ethics committee (CE4285). All patients provided written informed consent and authorized the use of their data.

## Results

Of 15,090 eligible patients, 13,215 were analyzed; 1,875 were excluded because of chronic kidney disease. Age was normally distributed, with a mean of 64 years (SD: 12). Most patients were men (75.7%). The main comorbidities were hypertension (66.0%), diabetes (20.2%), dyslipidemia (61.0%), smoking (45.0%), previous myocardial infarction (22.0%), COPD (4.7%), stroke (2.9%), and preoperative anemia (7.1%). Fourteen percent of patients were asymptomatic, 6.2% presented with heart failure, and 11.8% had ventricular dysfunction.

Regarding the surgical profile, most procedures were elective (69.6%). CABG was the most frequent surgery (47.9%), followed by valve surgery (23.2%) and combined procedures (12.6%) (Table 1).

**Table 1:** Clinical and demographic characteristics of the study population.

Characteristics	Total n (%)
Comorbidities	
Age, years (SD)	64 (12)
Male	10002 (75.7)
Hypertension	8740 (66)
Diabetes	2677 (20.2)
Dyslipidemia	8077 (61)
Smoking	6021 (45)
Previous MI	2918 (22)
COPD	624 (4.7)
Stroke	389 (2.9)
Preoperative anemia	933 (7.1)
Clinical presentation	
Asymptomatic	1855 (14)
Heart failure	815 (6.2)
Other characteristics	
Ventricular dysfunction	1558 (11.8)
CBP	6463 (50.1)
Elective surgery	9205 (69.6)
Reoperation	413 (3.1)
Type of surgery	
Coronary artery bypass grafting	6332 (47.9)
Valve surgery	3073 (23.2)
Combined procedure	1660 (12.6)

SD: standard deviation. n: number. MI: myocardial infarction. COPD: chronic obstructive pulmonary disease. CPB: cardiopulmonary bypass.

Overall, 7.3% of patients developed AKI. In-hospital mortality was 4.7%. Mortality was 22.0% among patients with AKI compared to 2.4% in those without postoperative renal failure (p<0.05). Dialysis was required in 3.6% of patients.

Univariate analysis included the main variables with clinical and prognostic relevance in the postoperative course of cardiac surgery (Table 2). Variables significant in univariate analysis were entered into the multivariate model to assess associations with the primary endpoint.

**Table 2:** Risk factors for acute kidney injury after cardiac surgery, bivariate analysis.

Variables	OR [95% CI]	p-value
Age	1.05 [1.04-1.06]	<0.001
Female	0.98 [0.78-1.23]	0.888
Urgent surgery	2.85 [2.41-3.37]	<0.001
CBP	1.39 [1.16-1.66]	<0.001
Previous MI	0.90 [0.74-1.11]	0.349
Previous PCI	1.08 [0.88-1.32]	0.444
Reoperation	1.41 [1.07-1.84]	0.013
COPD	1.33 [1.01-1.75]	0.041
Stroke	1.35 [0.97-1.87]	0.068
Preoperative anemia	1.35 [1.08-1.69]	0.007
Hypertension	0.93 [0.76-1.13]	0.496
Dyslipidemia	1.05 [0.88-1.26]	0.567
Smoking	1.04 [0.89-1.21]	0.586
Diabetes	1.04 [0.88-1.24]	0.593
Asymptomatic	0.69 [0.52-0.91]	0.009
MI	1.01 [0.75-1.35]	0.922
HF as clinical presentation	1.77 [1.41-2.23]	<0.001
Preoperative IABP	1.18 [0.78-1.80]	0.424
Sinus rhythm	0.83 [0.68-1.01]	0.074
Ventricular dysfunction	1.51 [1.24-1.85]	<0.001

OR: odds ratio. CI: confidence interval. CPB: cardiopulmonary bypass. MI: myocardial infarction. PCI: percutaneous coronary intervention. COPD: chronic obstructive pulmonary disease. HF: heart failure. IABP: intra-aortic balloon pump.

In multivariate analysis, preoperative predictors of AKI included age (OR: 1.05 [95% CI: 1.04-1.06]; p<0.001), COPD (OR: 1.33 [95% CI: 1.02-1.75]; p=0.040), and preoperative anemia (OR: 1.36 [95% CI: 1.09-1.69]; p=0.010). Heart failure as a clinical presentation (OR: 1.85 [95% CI: 1.47-2.31]; p<0.001) and ventricular dysfunction (OR: 1.50 [95% CI: 1.24-1.81]; p<0.001) were also associated with an increased risk (Table 3).

**Table 3:** Risk factors for acute kidney injury after cardiac surgery, multivariate analysis.

Variables	OR [95% CI]	p-value
Age	1.05 [1.04-1.06]	< 0.001
Urgent surgery	2.87 [2.44-3.37]	< 0.001
CPB	1.41 [1.19-1.66]	< 0.001
Reoperation	1.44 [1.10-1.88]	0.010
COPD	1.33 [1.02-1.75]	0.040
Preoperative anemia	1.36 [1.09-1.69]	0.010
Asymptomatic	0.68 [0.52-0.89]	0.010
HF as clinical presentation	1.85 [1.47-2.31]	< 0.001
Ventricular dysfunction	1.50 [1.24-1.81]	< 0.001

*The analysis was performed including all variables from Table 2.

OR: odds ratio. CI: confidence interval. CPB: cardiopulmonary bypass. COPD: chronic obstructive pulmonary disease. HF: heart failure.

Among surgical predictors, urgent surgery (OR: 2.87 [95% CI: 2.44-3.37]; p<0.001) and the use of CPB (OR: 1.41 [95% CI: 1.19-1.66]; p<0.001) were significant. As a postoperative predictor, the need for reoperation was also associated with AKI (OR: 1.44 [95% CI: 1.10-1.88]; p=0.010). Conversely, being asymptomatic (defined as patients requiring surgical intervention without prior symptoms related to their underlying disease) was identified as a protective factor associated with a lower risk of AKI (OR: 0.68 [95% CI: 0.52-0.89]; p=0.010) (Table 3).

## Discussion

In our study, the prevalence of AKI was relatively low (7.3%). We identified preoperative risk factors such as advanced age, comorbidities including COPD and anemia, ventricular dysfunction, and preoperative heart failure; intraoperative factors such as CPB and surgical urgency; and postoperative factors such as reoperation.

In contrast, registries including that of the American Heart Association (AHA) have reported higher incidences of AKI, reaching 20-30%. This discrepancy may be partly explained by the fact that 48% of CABG procedures in our cohort were performed off-pump, whereas CPB has consistently been identified as an independent predictor of PO-AKI. ^4^^,^^14^

Compared with the CONAREC registry, which reported an overall prevalence of 13.3%, our incidence was also lower, probably reflecting that our institution is a high-volume, single-specialty cardiovascular center performing more than 700 surgeries annually. Similarly, the AKI incidence after CABG was 5.3% in our series, lower than the 12.5% reported in the ARGEN-CCV registry, in which around 50% of CABG procedures were performed with CPB. ^8^

PO-AKI in cardiac surgery is known to increase morbidity and mortality. International registries report a mortality rate of 10% in the short term and 30% in the long term; in our cohort, in-hospital mortality among patients with AKI reached 22%. ^4^

AKI development is multifactorial, with predictors including advanced age, comorbidities such as diabetes and hypertension, chronic kidney disease, and exposure to nephrotoxic agents. Intraoperative factors such as the duration of CPB, prolonged hypotension, and the type of surgical procedure also play key roles. Predictive models such as EuroSCORE have been developed to estimate postoperative risk, including AKI. ^11^^,^^15^^-^^17^

Our findings are consistent with previous reports highlighting advanced age, surgical urgency, and comorbidities as major risk factors for AKI after cardiac surgery. ^5^^,^^10^^)^ The association between CPB and AKI is well documented, reinforcing the need for perioperative optimization strategies to mitigate this effect. ^11^ The observation that heart failure and ventricular dysfunction increase risk further emphasizes the need for stricter perioperative management in these patients.

Recent studies have proposed novel strategies for prediction, prevention, and management, including biomarkers such as cystatin C and neutrophil gelatinase-associated lipocalin (NGAL). ^5^ These biomarkers show promise for early detection and risk stratification, although clinical implementation requires further evidence.

The pathophysiology of cardiac surgery-associated AKI is complex, with ischemia-reperfusion injury during and after CPB playing a central role. This triggers inflammation, oxidative stress, and renal cell death, leading to complement activation and secondary damage. ^18^^-^^21^

Ongoing clinical trials are evaluating targeted therapies, such as inhibition of the P2X9 receptor or blockade of complement C5 with ravulizumab. ^12^ Other studies suggest that intravenous amino acids may improve renal perfusion and functional reserve, potentially reducing AKI incidence in selected patients. ^22^

Additional perioperative management strategies are being developed, including hemodynamic optimization, renal preservation techniques, and adjustment of nephrotoxic drug regimens. Interventions aimed at improving preoperative conditions might also reduce AKI incidence and impact in cardiac surgery. ^23^

This study has limitations. First, its observational design entails risks of selection, information, and confounding bias since unmeasured variables may have influenced outcomes. To minimize systematic error, we applied an appropriate design and multivariate logistic regression. Second, this was a single-center study from a high-complexity cardiovascular institution, which limits generalizability to the wider Argentine context. Finally, the study period spanned 20 years, during which surgical practices evolved. However, PO-AKI management did not change substantially, and no novel therapies emerged during this time to alter prognosis.

Despite these limitations, further efforts should focus on developing new strategies and pharmacological therapies specifically targeting PO-AKI in cardiac surgery.

In conclusion, AKI prevalence in our cohort was low (7.3%). Independent predictors included age, CPB, and surgical urgency. These findings underscore the importance of identifying risk factors and implementing tailored preventive strategies. The integration of novel diagnostic tools and perioperative management approaches could substantially improve outcomes for these patients.
